# No Increased Risk for Primary Osteoarthritis in Liver Cirrhosis – A Danish Nationwide Cohort Study

**DOI:** 10.1371/journal.pone.0167134

**Published:** 2016-11-29

**Authors:** Thomas Deleuran, Hendrik Vilstrup, Søren Overgaard, Peter Jepsen

**Affiliations:** 1 Department of Hepatology and Gastroenterology, Aarhus University Hospital, Aarhus, Denmark; 2 Department of Clinical Epidemiology, Aarhus University Hospital, Aarhus, Denmark; 3 Department of Orthopedic Surgery and Traumatology, Odense University Hospital, Institute of Clinical Research, University of Southern Denmark, Denmark; Universidad de Navarra, SPAIN

## Abstract

**Objective:**

Chronic synovial inflammation causes primary osteoarthritis, but it is unknown whether chronic systemic inflammation does, too. Patients with cirrhosis have chronic systemic inflammation and therefore we examined the association between cirrhosis and primary osteoarthritis of the hip and knee.

**Methods:**

In Danish healthcare databases we identified all residents over 60 years diagnosed with cirrhosis in 1994–2011, and for each of them we sampled five age- and gender-matched reference persons from the general population. We excluded everyone with risk factors for secondary osteoarthritis and computed incidence rates of primary osteoarthritis of the hip or knee. We used stratified Cox regression to estimate the hazard ratios of primary osteoarthritis for cirrhosis patients vs. reference persons in strata defined by gender, age, cirrhosis etiology, and ascites vs. no ascites. We also computed separate HRs for primary osteoarthritis of the hips or knees.

**Results:**

We identified 10,049 cirrhosis patients. Their median age was 67 years, and 65% were men. Among the cirrhosis patients the crude incidence rate of primary osteoarthritis was 8.40 (95% CI: 7.30–9.63) per 1000 person-years. The rate was similar in the reference persons: 8.76 (95% CI: 8.43–9.12) per 1000 person-years. Accordingly, the hazard ratio for primary osteoarthritis for cirrhosis patients vs. reference persons was 0.99 (95% CI: 0.85–1.16), and we found the same null association in all patient strata and in both joints.

**Conclusion:**

Cirrhosis, and thus chronic systemic inflammation, is not a risk factor for primary osteoarthritis.

## Introduction

Osteoarthritis causes pain, stiffness, and reduced physical activity and quality of life [1]. Secondary osteoarthritis has a well-known etiology such as sequelae to trauma, congenital abnormalities, infection, or rheumatoid arthritis, whereas primary osteoarthritis is defined by absence of such previous pathology. Like old age and biomechanical stress, localized joint inflammation is a risk factor for primary osteoarthritis [2], and we hypothesized that also chronic systemic inflammation increases the risk of primary osteoarthritis [3]. This possibility is supported by studies linking raised serum markers for inflammation with radiographic changes, symptoms, and symptom progression in primary osteoarthritis [4, 5]. Cirrhosis is the end-stage of all chronic liver diseases and characterized by chronic systemic inflammation [6]. Thus, cirrhosis patients offer an opportunity to study chronic systemic inflammation as a risk factor for primary osteoarthritis, and this has not been examined before. Given this background, our objective was to examine cirrhosis as a risk factor for primary osteoarthritis of the hip or knee.

## Patients and methods

### Data sources

We performed a nationwide registry-based historical matched cohort study set in the country of Denmark, which has 5.6 million inhabitants. All Danish residents are provided universal, tax-paid access to hospitals. The Danish National Patient Registry (NPR) is a nationwide registry that covers admissions to non-psychiatric hospitals after 1977, and outpatient and emergency room visits after 1995. The data includes relevant dates and discharge diagnoses coded in accordance with the International Classification of Diseases, edition 10 (ICD-10) from 1994 and the ICD-8 before that [7]. The Danish Hip Arthroplasty Registry (DHR) covers all total hip arthroplasties (THA) in Denmark since 1 January 1995, and the Danish Knee Arthroplasty Registry (DKR) covers all total knee arthroplasties (TKA) in Denmark since 1 January 1997 [8, 9]. These clinical databases include the indication for arthroplasty (primary osteoarthritis, or other indication) [8, 9]. The Danish Central Office of Civil Registration continuously monitors Danish residents’ vital status including dates of emigration or death and issues a unique personal identifier to all residents in Denmark at birth or immigration. This number enables linkage of individual-level data between the NPR, the DHR/DKR, and the civil registration system [10]. The study was approved by the Danish Data Protection Agency (Permission number: 2012-41-0140).

### Cirrhosis patients and reference persons

Primary osteoarthritis mainly affects the elderly, so we restricted the study to people aged 60 years or older. First, we identified all Danish citizens with a hospital discharge diagnosis of alcoholic cirrhosis (ICD-10: K70.3, K70.4) or unspecified cirrhosis (ICD-10: K74.6) between 1994 and 2011 in the Danish National Patient Registry (NPR). We defined the ‘index date’ as the date of their first cirrhosis diagnosis, and identified cirrhosis patients aged 60 years or more on the index date. Among them, we excluded cirrhosis patients who before the index date had a diagnosis for primary osteoarthritis (ICD-10: M16.x, M17.x, ICD-8: 713.xx) or for a condition predisposing to secondary osteoarthritis (S1 Table), or who had already undergone THA/TKA. All the remaining cirrhosis patients were included.

Cirrhosis patients with ascites may have an increased load on weight bearing joints. Therefore, the cirrhosis patients were subdivided into those who on or before the index date had a diagnosis code for ascites (ICD-8: 785.39, ICD-10: R18.9) or a procedure code for a paracentesis (ICD-10: KTJA10) and those without. We matched the cirrhosis patients 1:5 on age, gender and birth year to persons without cirrhosis from the general Danish population, using risk set sampling [11], and these reference persons were given the same index date as their corresponding cirrhosis patient. Subsequently, we applied the same exclusion criteria to the reference persons as to the cirrhosis patients, so not all cirrhosis patients were matched 1:5 in the analysis (60% were matched 1:5, 32% were matched 1:4, and the remaining 8% had 3 or fewer reference persons).

### Primary osteoarthritis

We examined two outcomes: First, time to a first-time hospital diagnosis for primary osteoarthritis of the hip or the knee (ICD-10: M16.0, M16.1 or M17.0, M17.1) according to the NPR. Second, in order to ensure that everyone had equally severe osteoarthritis and that diagnoses were correct, we defined a composite outcome: time to a diagnosis for osteoarthritis of the hip or knee in the National Patient Registry *and* a subsequent THA/TKA for primary osteoarthritis at the same site (hip/knee) according to the DHR/DKR.

### Statistical analysis

We followed the cirrhosis patients and the reference persons from the index date to the date of their first diagnosis for primary osteoarthritis, death, or end of follow-up on 31 December 2011. When we analyzed the composite outcome (NPR diagnosis of primary osteoarthritis *and* a subsequent THA/TKA), we followed the cirrhosis patients and their reference population to the date of THA/TKA. We computed crude incidence rates of primary osteoarthritis of the hip or knee for cirrhosis patients and reference persons. Cox regression was used to estimate the hazard ratio (HR) for primary osteoarthritis for cirrhosis patients vs. reference persons. We computed HRs within subgroups defined by gender, age on the index date (60–69, 70–79, >79 years), alcoholic cirrhosis and unspecified cirrhosis, and for cirrhosis patients with and without ascites. We also computed separate HRs for primary osteoarthritis of the hip or knee, and for the composite outcome. Smoking may protect against primary osteoarthritis [12], so we adjusted the HRs for previous hospital admissions for chronic obstructive pulmonary disease (ICD-8: 490.xx, 491.xx, 492.xx; ICD-10: J43.x, J44.x). All statistical analyses were performed using Stata version 12.1 (StataCorp, College Station, Texas) and R version 2.14 [13].

## Results

We included 10,049 cirrhosis patients and 44,370 matched reference persons. Their median age was 67 years, 65% were male, 66% had alcoholic cirrhosis, and 33% had ascites; 10.5% of the cirrhosis patients and 4.5% of the reference persons had chronic obstructive pulmonary disease. A total of 208 cirrhosis patients and 2,490 reference persons were diagnosed with primary osteoarthritis of the hip or knee during the follow-up. Among the cirrhosis patients the crude incidence rate of primary osteoarthritis was 8.40 (95% CI: 7.30–9.63) per 1000 person-years compared with 8.76 (95% CI: 8.43–9.12) per 1000 person-years among the reference persons; the incidence rates were similar in cirrhosis patients and reference persons in all age groups (Table 1).

**Table 1:** Incidence rate (IR) of primary osteoarthritis of the hip or knee in Danish cirrhosis patients and age- and gender-matched reference persons from the general population.

	Age group (years)	Number of patients diagnosed with primary osteoarthritis	Size of population	Follow-up time (years)	IR per 1000 person years
Cirrhosis patients	60–69	152	6,634	18,462	8.23 (95% CI: 6.97–9.65)
	70–79	50	2,746	5,515	9.06 (95% CI: 6.72–11.9)
	80+	6	669	771	7.78 (95% CI: 2.86–16.9)
	Total	208	10,049	24,748	8.40 (95% CI: 7.30–9.63)
Reference persons	60–69	1,739	29,658	199,273	8.73 (95% CI: 8.32–9.14)
	70–79	679	11,914	72,424	9.37 (95% CI: 8.68–10.1)
	80+	72	2,798	12,259	5.87 (95% CI: 4.59–7.39)
	Total	2,490	44,370	289,015	8.76 (95% CI: 8.43–9.12)

As we anticipated, chronic obstructive pulmonary disease was associated with a reduced rate of primary osteoarthritis (HR for persons with and without chronic obstructive pulmonary disease = 0.69, 95% CI: 0.53–0.90), but even when we adjusted the osteoarthritis rates for chronic obstructive pulmonary disease’s confounding effect, cirrhosis remained unassociated with primary osteoarthritis (HR ratio for cirrhosis vs. reference persons = 0.99, 95% CI: 0.85–1.16). This HR was similar across gender and age groups; similar for cirrhosis patients with or without ascites; similar for patients with alcoholic cirrhosis or unspecified cirrhosis; and similar for primary osteoarthritis of the hip or the knee (Table 2).

**Table 2:** Hazard ratio (HR) for primary osteoarthritis for cirrhosis patients vs. reference persons by gender, age on index date, ascites, cirrhosis etiology, and site.

	HR*
Overall	0.99 (95% CI: 0.85–1.16)
Gender	
Males	0.97 (95% CI: 0.79–1.20)
Females	1.02 (95% CI: 0.82–1.28)
Age group	
60–69	1.02 (95% CI: 0.85–1.22)
70–79	0.94 (95% CI: 0.69–1.29)
80+	0.93 (95% CI: 0.38–2.31)
Ascites	
Yes	0.80 (95% CI: 0.60–1.07)
No	0.97 (95% CI: 0.84–1.14)
Cirrhosis etiology	
Alcoholic cirrhosis	0.90 (95% CI: 0.74–1.09)
Unspecified cirrhosis	1.18 (95% CI: 0.93–1.51)
Site	
Hip	1.17 (95% CI: 0.95–1.44)
Knee	0.92 (95% CI: 0.74–1.14)
Composite outcome†	0.78 (95% CI: 0.60–1.01)

*Adjusted for chronic obstructive pulmonary disease

†A diagnosis for primary osteoarthritis of the hip or knee *and* a subsequent THA/TKA for primary osteoarthritis.

However, the HR for the composite outcome for cirrhosis patients vs. reference persons was 0.78 (95% CI: 0.60–1.01), which may indicate that even though cirrhosis patients and reference persons have a similar rate of osteoarthritis, cirrhosis patients are less likely to undergo THA/TKA for primary osteoarthritis.

## Discussion

In this nationwide cohort study we found that cirrhosis was not a risk factor for primary osteoarthritis, and the narrow confidence interval ranging from 0.85 to 1.16 essentially rules out a clinically relevant association. Moreover, we found a similar effect of cirrhosis on the risk for primary osteoarthritis in subgroups defined by age, gender, alcoholic vs. unspecified cirrhosis, and for cirrhosis patients with and without ascites.

The strength of our study is its foundation in the Danish healthcare data which enables precise estimates, complete follow-up, and a population-based design. The study population was based on *hospitalized* patients with cirrhosis. Consequently, persons with cirrhosis that for various reasons were treated outside hospitals were not included. Moreover, 6% of patients with biopsy-verified cirrhosis did not have a discharge diagnosis for cirrhosis [14]. Altogether, our study might overlook an unknown proportion of Danish residents with cirrhosis, but these patients probably have less severe cirrhosis compared with hospitalized cirrhosis patients. Therefore, we find it unlikely that their risk of primary osteoarthritis is different from hospitalized cirrhosis patients.

Cirrhosis diagnoses recorded in the NPR were confirmed by biopsy or clinical evaluation in 85% of cases [14], and we find it unlikely that the 15% without cirrhosis in our patient cohort caused us to miss an existing association; that could only happen if all those 15% possessed a factor that confers a greatly reduced (or increased) risk of primary osteoarthritis. The NPR diagnoses for primary osteoarthritis have never been validated, but we corroborated our results by combining diagnoses for primary osteoarthritis in the NPR with data on the indication for arthroplasty from the DHR/DKR. The validity of the indication ‘primary osteoarthritis’ in the DHR is 85% [9], and we assume that the validity of this indication is similar in the DKR. We expect the validity to be the same for cirrhosis patients and reference persons. So, we do not believe that inadequacies of our data sources impaired the reliability of our results.

The most important risk factor for primary osteoarthritis is old age, and this potential confounder was eliminated by the matching. The use of hospital diagnosis codes for chronic obstructive pulmonary disease as an indicator for smoking will underestimate its prevalence. So if cirrhosis patients’ higher prevalence of smoking decreases their risk of primary osteoarthritis, we may underestimate the hazard ratio slightly. Danish cirrhosis patients have a low employment rate [15], and socioeconomic deprivation increases the risk the risk for primary osteoarthritis [16]. Thus, the lack of adjustment for socioeconomic factors will probably cause to overestimate the hazard ratio. Mechanical stress is also a potential confounder. We had no data on body mass index (BMI), and cirrhosis patients’ fluctuating volume of ascites makes their BMI a poor marker of mechanical stress; and notably, ascites did not alter the association between cirrhosis and primary osteoarthritis. Ethnicity is an unlikely confounder because the Danish population is 95% Caucasian [17]. Alcohol consumption is a possible confounder, but we found a similar association when we divided our analyses into patients with alcoholic cirrhosis and patients with unspecified cirrhosis. So, although we cannot exclude uncontrolled confounding from smoking, BMI, socioeconomic factors, ethnicity and alcohol completely, we do not believe that it explained our results.

From a clinical point of view, the lack of an increased risk for osteoarthritis in cirrhosis is valuable information, because we have shown that hip- and knee arthroplasty in such patients carry an increased risk for postoperative complications [18–20]. The increased risk of complications may imply that orthopedic surgeons hesitate to perform arthroplasty in cirrhosis patients, and that is the best explanation to the slightly lower rate of arthroplasty for osteoarthritis in the cirrhosis patients than in the reference persons.

The increased risk of complications also raises the possibility that cirrhosis patients are less likely to be referred to work-up for primary osteoarthritis than reference patients without cirrhosis, because they are deemed unfit for arthroplasty surgery. This scenario might produce a referral bias that would cause us to underestimate the true association between cirrhosis and primary osteoarthritis. However, physiotherapy offers an alternative to surgery and is not contraindicated in cirrhosis patients, so it is always clinically meaningful to refer cirrhosis patients to work-up for joint pain. Moreover, we find it unlikely that the effect of a referral bias nullifies a true association between cirrhosis and primary osteoarthritis in the main analysis *and* in all strata defined by age and gender. All in all, we acknowledge that a referral bias might exist, but we maintain our conclusion that cirrhosis not is a risk factor for primary osteoarthritis.

There is strong evidence that cirrhosis patients suffer from chronic systemic inflammation [6], so the absence of an association between cirrhosis and primary osteoarthritis refutes our hypothesis that chronic systemic inflammation causes primary osteoarthritis. Previous studies have found that biomarkers for inflammation (Interleukin-6 and Tumor Necrosis Factor α) are elevated in alcoholic cirrhosis patients and predict increasing knee pain in patients with primary osteoarthritis [4, 5, 21]. One way to unite those findings with ours is to posit that it takes decades of systemic inflammation to develop primary osteoarthritis. Cirrhosis patients do not survive long enough to settle that possibility [22], but patients with less life-threatening chronic inflammatory diseases, such as asthma or inflammatory bowel disease, are not good models, either; their systemic inflammation is only active during exacerbations.

In conclusion, cirrhosis was not associated with primary osteoarthritis of the hip and knee, and this null association was also present when we analyzed subgroups defined by age, gender, alcohol vs. unspecified cirrhosis, and with vs. without ascites. This result suggests that chronic systemic inflammation does not have an important role in primary osteoarthritis.

## Supporting Information

S1 Table Causes of secondary osteoarthritis of the hip or knee (DOCX)
