# Supplementary material for: No Increased Risk for Primary Osteoarthritis in Liver Cirrhosis – A Danish Nationwide Cohort Study
Source: PLoS One. 2016 Nov 29;11(11):e0167134. doi: 10.1371/journal.pone.0167134 (PMC5127570; doi:10.1371/journal.pone.0167134)
Supplement: S1 Table — (DOCX) [file pone.0167134.s001.docx]

# S1 Table

| **Disorder** | **ICD-8** | **ICD-10** |
| --- | --- | --- |
| Avascular necrosis | - | M87.0 |
| Calve-Legg-Perthes | 722.11 | M91.1 |
| Congenital hip dislocation | 755.69 | Q65.x |
| Epifysiolysis | 722.10 | M93.0 |
| Fracture of distal femur, patella, or proximal tibia | 821.xx,822.xx,823.xx | S82.0,S82.1 |
| Hip fracture, acetabulum fracture | 820.xx | S32.4,S32.5,S72.x |
| Hip dislocation, knee dislocation | 835.xx,836.xx | S73.x, S83.x |
| Rheumatoid arthritis, other arthritis | 710.xx,711.xx,712.xx,714.xx,715.xx | M0x.x,M10.x,M11.x,M12.x,M13.x,M14.x |
